# Molecular Insights and Antibiotic Resistance of *Proteus mirabilis* from Retail Meat Sources in Iran

**DOI:** 10.1002/fsn3.71832

**Published:** 2026-05-13

**Authors:** Shahin Kouhi Kamali, Elahe Tajbakhsh, Faham Khamsipour, Hassan Momtaz

**Affiliations:** ^1^ Department of Microbiology ShK.C., Islamic Azad University Shahrekord Iran; ^2^ Halal Research Center of the Islamic Republic of Iran (IRI), Iran Food and Drug Administration Ministry of Health and Medical Education Tehran Iran

**Keywords:** antibiotic resistance, biofilm, ERIC‐PCR, meat contamination, *P. mirabilis*, REP‐PCR, virulence genes

## Abstract

*Proteus mirabilis* ( *P. mirabilis* ) is an emerging foodborne pathogen that poses a risk to both veterinary and human health because of its virulence factors and multidrug resistance (MDR) capability. This study aimed to investigate the molecular characteristics, virulence genes, antibiotic resistance, and genetic diversity of *P. mirabilis* isolated from red meat and poultry in Shahrekord, Iran. A total of 480 meat samples (red meat and poultry) were examined for *P. mirabilis* contamination using biochemical and molecular methods. Biofilm formation was evaluated using the microtiter plate assay. Antibiotic susceptibility was determined by the disk diffusion method. Extended‐spectrum *β*‐lactamase (ESBL) production was detected by the combined disk test. Resistance and virulence genes were identified by PCR. Genetic diversity was analyzed using Enterobacterial Repetitive Intergenic Consensus‐Polymerase Chain Reaction (ERIC‐PCR) and Repetitive Extragenic Palindromic‐Polymerase Chain Reaction (REP‐PCR). Out of 480 samples, 92 (19.16%) were positive for *P. mirabilis* , with the highest prevalence in veal (25%) and chicken (27.5%). Over 79% of isolates were strong biofilm producers. The highest resistance rates were observed for nitrofurantoin (70.8%) and trimethoprim‐sulfamethoxazole (79.5%). ESBL‐producing isolates carried *bla*CTX‐M and *bla*TEM genes, whereas *bla*SHV was less frequent. The most common resistance genes were *qnr*A, *qnr*B, *tet*A, *tet*B, and *sul1*. Virulence genes such as *zap*A, *hpm*A, *mrp*A, and *hly*A were detected in most isolates. ERIC‐PCR and REP‐PCR revealed high genetic diversity among isolates with 75%–100% similarity for red meat isolates and 51%–100% for poultry isolates. *P. mirabilis* isolates from red meat and poultry in Shahrekord showed high prevalence of MDR and virulence genes, indicating potential risks to food safety and public health. Continuous monitoring and stricter control measures in meat production are necessary to prevent the spread of resistant and virulent strains.

## Graphical abstract

Isolation and characterization of antibiotic‐resistant *Proteus mirabilis* from retail meat in Iran. The workflow illustrates the screening of meat samples, genomic identification of *P. mirabilis*, and molecular analysis of resistance genes.

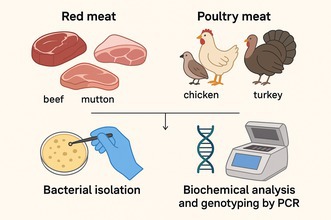

## 1 Introduction

In veterinary medicine, the bacterium *P. mirabilis* is increasingly recognized as a potential zoonotic and food‐borne pathogen, able to enter the animal‐food‐human interface via meat and poultry products. As animal‐derived foods serve as major protein sources, contamination of red meat and poultry with *P. mirabilis* raises concerns for veterinarians, food‐safety regulators, and clinicians alike. This pathogen's presence in meats suggests a need for animal‐health surveillance and cross‐sectoral One Health vigilance (Zhao et al. 2022). From a veterinary‐microbiology perspective, *P. mirabilis* resides in the gastrointestinal tracts of animals, thrives in farm and processing environments, and shows the ability to persist on surfaces and in biofilms. Its survival traits make it more than a simple commensal: it may act as a reservoir of antimicrobial resistance (AMR) and virulence factors in livestock systems (O'Hara and Miller 2000; Girlich et al. 2020). The use of antimicrobials in animal husbandry—whether for therapeutic, metaphylactic or growth‐promoting purposes—applies selection pressure favoring resistant bacteria. In poultry and red meat production, this can lead to the emergence of multidrug‐resistant (MDR) *P. mirabilis* strains that may enter the food chain. Genetics of acquired resistance in *P. mirabilis* include ESBLs, AmpC cephalosporinases, and carbapenem's, which complicate veterinary and public‐health interventions (Pitout and Laupland 1998; Naas et al. 2003; De Champs et al. 2000). In the slaughter‐to‐retail continuum, veterinarians and inspectors must recognize multiple contamination points: during slaughter, evisceration, carcass handling, transport, and storage. *P. mirabilis* —with its biofilm‐forming ability and resistance determinants—can survive in abattoirs and meat‐processing plants, posing cross‐contamination risks from animal to human foods (Wong et al. 2022; Boolchandani et al. 2019a; 2019b).

Molecular characterization tools such as ERIC‐PCR, REP‐PCR, and whole‐genome sequencing empower veterinary microbiologists to evaluate clonal spread, source attribution, and resistance gene dissemination among animal, food, and human isolates of *P. mirabilis* . The role of such tools in livestock surveillance is increasingly highlighted (Chalmers, Xie, and Wang 2023; Chalmers, McAllister, et al. 2023; Li et al. 2022). Epidemiological studies in the meat and poultry sectors indicate that chicken meat more frequently harbors *P. mirabilis* carrying extended‐spectrum *β*‐lactamase (ESBL) and AmpC genes compared with beef, underscoring the need for specific surveillance in poultry‐derived products in veterinary food‐safety programmes (Durso et al. 2023; Li et al. 2022). From the veterinary public‐health view, red meat and poultry are consumed widely in Iran; thus, contamination of these products with MDR *P. mirabilis* represents a direct animal‐food‐human risk that warrants assessment through region‐specific studies focusing on origin, prevalence, molecular traits, and resistance profiles.

In the region of Shahrekord, Iran—characterized by livestock and poultry production—veterinary services and food‐safety authorities have limited data on *P. mirabilis* in meat products. A dedicated study addressing its molecular traits and resistance patterns will bridge an important knowledge gap for local veterinary public‐health professional practice. A comprehensive veterinary‐microbiology study of *P. mirabilis* in meat should integrate phenotypic antibiotic‐susceptibility testing, detection of resistance and virulence genes, biofilm‐formation assessment and molecular typing strategies, thus enabling veterinary practitioners and food‐safety inspectors to derive actionable control measures at the farm, slaughter and retail levels. The present work on isolates from Shahrekord, Iran, aims to provide evidence on prevalence, molecular characteristics, and resistance profiles of this organism in meat products, thereby supporting veterinary public‐health strategies and contributing to the One Health agenda.

## 2 Materials and Methods

### 2.1 Study Design, Study Population, and Sampling Units

The study population comprised samples of red meat (beef, mutton, and veal) and poultry meat (chicken, quail, and turkey) collected from retail outlets in Shahrekord, Iran. Sampling was performed during all four seasons of 2022–2023 (1401 SH) to cover potential seasonal variations in contamination rates. A total of 480 samples were collected (80 per meat type), as shown in Table 1. The sample size was estimated using a 35% average prevalence of *P. mirabilis* reported in previous studies, applying the formula *n = z* ^*2*^ *pq/d* ^*2*^. This was an applied, descriptive, and cross‐sectional study designed to identify and characterize *P. mirabilis* isolates from different meat sources, determine their antibiotic resistance profiles, and perform molecular genotyping.

**TABLE 1:** The study population comprised samples of red meat.

Meat type	Number of samples
Beef	80
Mutton	80
Veal	80
Chicken	80
Quail	80
Turkey	80
Total	480

### 2.2 Sampling and Transportation

Samples were obtained by random stratified sampling proportional to volume from butcheries and poultry shops across Shahrekord County. Each sample (~25–50 g) was aseptically collected, placed in sterile polyethylene bags, and transported in ice boxes at 4°C to the Microbiology Research Laboratory, Islamic Azad University, Shahrekord Branch, within 2 h of collection.

### 2.3 Sample Preparation and Bacterial Isolation

Under a laminar‐flow hood, meat samples were aseptically minced using sterile scalpels. Ten grams of each sample were homogenized in 90 mL of Tryptone Soy Broth (TSB; Hi‐Media, India) using a stomacher for 2 min, followed by incubation at 37°C for 24 h. Subsequently, 300 μL of the broth culture was streaked on Xylose Lysine Deoxycholate (XLD) agar (Merck, Germany) and incubated at 37°C for 24 h. Black colonies suspected of *P. mirabilis* were purified and identified on the basis of morphological characteristics, swarming motility, and standard biochemical tests including IMViC, urease, H₂S production, maltose fermentation, and ornithine decarboxylase reactions. *P. mirabilis ATCC 12453* and *Klebsiella pneumoniae ATCC 700603* were used as positive and negative quality‐control strains, respectively.

### 2.4 Antibiotic Susceptibility Testing

Antibiotic susceptibility of the confirmed isolates was determined by the Kirby–Bauer disk diffusion method (CLSI 2022) on Mueller–Hinton agar (Merck, Germany), according to CLSI (2022) guidelines. The antibiotic disks (Padtan Teb, Iran) included: Trimethoprim–sulfamethoxazole (SXT 25 μg), Amikacin (AN 30 μg), Gentamicin (GM 10 μg), Nitrofurantoin (FM 300 μg), Cefotaxime (CTX 30 μg), Ceftazidime (CAZ 30 μg), Ceftriaxone (CRO 30 μg), Cephalothin (CF 30 μg), Ciprofloxacin (CP 5 μg), Norfloxacin (NOR 10 μg), Nalidixic acid (NA 30 μg), Tetracycline (TE 30 μg). Interpretation of inhibition zones followed the CLSI 2022 criteria. *E. coli ATCC 25922* was used as the control strain.

### 2.5 Preparation of 0.5 McFarland Standard and Bacterial Suspension

The 0.5 McFarland standard was prepared by mixing 0.5 mL of 0.048 M BaCl_2_ with 99.5 mL of 0.36 N H_2_SO_4_, corresponding to 1.5 × 10^8^ CFU/mL. The optical density was verified at 625 nm (OD = 0.08–0.13). For inoculum preparation, 3–5 colonies from 24 h cultures on blood agar were suspended in sterile saline and adjusted to match the 0.5 McFarland turbidity standard. A sterile swab was used to uniformly inoculate Mueller–Hinton agar plates, and antibiotic disks were applied at 1.5 cm intervals. Plates were incubated at 37°C for 24 h, and inhibition zones were measured to the nearest millimeter.

### 2.6 Phenotypic Detection of ESBL‐Producing Strains

Screening for extended‐spectrum *β*‐lactamase (ESBL) production was performed on isolates resistant to one or more third‐generation cephalosporins (cefotaxime, ceftazidime, ceftriaxone, or cephalothin). Confirmation was carried out using the combined disk test (CDT) with and without clavulanic acid. An increase ≥ 5 mm in the inhibition zone diameter around cephalosporin + clavulanic acid disks compared with cephalosporin alone indicated ESBL production. *K. pneumoniae ATCC 700603* served as a positive control.

### 2.7 Biofilm Formation Assay

Biofilm formation was evaluated by the 96‐well microtiter plate assay. Each isolate was cultured overnight in TSB, then diluted 1:10 in TSB supplemented with 0.25% glucose. Aliquots (200 μL) were transferred to sterile flat‐bottom 96‐well plates and incubated at 37°C for 24 h. Wells were washed three times with sterile saline, stained with 0.9% crystal violet for 15 min, washed, and fixed with 95% ethanol. The optical density (OD) was measured at 630 nm using a microplate reader. Biofilm formation strength was classified as follows: Strong: OD > 4 × ODc, Moderate: 2 × ODc < OD ≤ 4 × ODc, Weak: ODc < OD ≤ 2 × ODc, Negative: OD ≤ ODc. *K. pneumoniae ATCC 1705* was used as a positive control.

### 2.8 DNA Extraction and Quality Assessment

Genomic DNA was extracted using a commercial DNA extraction kit (SinaClon, Iran) following the manufacturer's protocol. DNA purity and concentration were assessed spectrophotometrically at 260/280 nm, and integrity was checked by 1% agarose gel electrophoresis. Samples with OD 260/280 ratios between 1.8 and 2.0 were considered suitable for PCR.

### 2.9 Molecular Confirmation of *P. mirabilis*

Confirmation of *P. mirabilis* isolates was performed by PCR amplification of the ureR gene using specific primers (F: CCGGAACAGAAGTTGTCGCTGGA; R: GGGCTCTCCTACCGACTTGATC), producing a 359 bp amplicon. PCR reactions (25 μL) contained 1 μL DNA template (~50 ng), 1× PCR buffer, 0.2 mM dNTPs, 1.5 mM MgCl₂, 1 μM of each primer, and 1 U Taq DNA polymerase. Cycling conditions: 95°C 5 min; 35 cycles of 94°C 1 min, 58°C 1 min, 72°C 1 min; final extension 72°C 10 min. Products were visualized on 2% agarose gels under UV light.

### 2.10 Detection of Antibiotic‐Resistance and Virulence Genes

PCR assays were performed to detect resistance genes (*qnrA, qnrB, qnrS, tetA, tetB, sul1, blaCTX‐M, blaSHV, blaTEM, aac(3)IIa*, and *ant(3)Ia*) and virulence genes (*hpmA, hpmB, mrpA, hlyA, zapA, pmfA*, and *atfA*). Primer sequences and expected amplicon sizes were adapted from published references and confirmed via NCBI BLAST. Each 25 μL reaction included 1 μL DNA, 0.2 mM dNTPs, 1.5 mM MgCl_2_, 1 μM of each primer, and 1.5 U Taq polymerase. Cycling conditions varied depending on primer annealing temperature (58°C–60°C). Amplicons were resolved on 2% agarose gels and photographed using a Gel Doc system (Zhao et al. 2022).

### 2.11 Genotyping by ERIC‐PCR and REP‐PCR

Genetic diversity of *P. mirabilis* isolates was analyzed using ERIC‐PCR and REP‐PCR. ERIC‐PCR primers: ERIC‐1 (ATGTAAGCTCCTGGGGATTCAC) and ERIC‐2 (AAGTAAGTGACTGGGGTGAGCG). REP‐PCR primers: REP‐1 (III CGI CATC IGG C) and REP‐2 (ICG ICT TATC IGG CCT AC). PCR products were electrophoresed on 2% agarose gels, and banding patterns were analyzed using GelJ software. Clustering was based on Dice similarity coefficients and UPGMA algorithms, with ≥ 80% similarity considered as one genotype.

### 2.12 Data Analysis

Statistical analyses were performed using SPSS v18.0. Associations between meat type, season, antibiotic resistance patterns, and gene presence were assessed using Chi‐square and Fisher's exact tests with a significance level of *p ≤ 0.05*. Dendrograms representing genetic similarity among isolates were generated using UPGMA analysis in GelJ software.

## 3 Results

### 3.1 Microbiological Analysis

Out of 480 meat samples analyzed, 92 (19.16%) were positive for *P. mirabilis* . Among 240 red‐meat samples, 48 (20%) were contaminated, whereas 44 (18.33%) of 240 poultry samples were positive. The highest prevalence among red meat was recorded in veal (25%), followed by mutton (22.5%) and beef (12.5%) (Table 2). Among poultry, the highest contamination was observed in chicken meat (27.5%), followed by quail (15%) and turkey (12.5%) (Table 3). A statistically significant association was found between sample type and *P. mirabilis* contamination (χ^2^ test, *p* < 0.05).

**TABLE 2:** Frequency of *P. mirabilis* isolated from red‐meat samples.

Sample type	No. tested	Positive	% positive
Beef	80	10	12.5
Mutton	80	18	22.5
Veal	80	20	25
Total	240	48	20

**TABLE 3:** Frequency of *P. mirabilis* isolated from poultry meat.

Sample type	No. tested	Positive	% positive
Chicken	80	22	27.5
Quail	80	12	15
Turkey	80	10	12.5
Total	240	44	18.33

Biochemical identification confirmed *P. mirabilis* as a Gram‐negative, motile, urease‐positive *bacillus*, showing positive MR, VP, citrate, H_2_S, and ornithine decarboxylase reactions and negative indole, maltose, and sucrose fermentation tests (Table 4).

**TABLE 4:** Biochemical profile of *P. mirabilis* isolates.

Test	Indole	MR	VP	Citrate	Maltose	Sucrose	H_2_S	Ornithine decarboxylase	ureR gene (359 bp)
*P. mirabilis*	−	+	+	+	−	−	+	+	+

### 3.2 DNA Extraction and Confirmation

DNA extracted from all *P. mirabilis* isolates produced distinct, intact genomic bands on 1% agarose gel electrophoresis, confirming DNA purity and suitability for PCR. Amplification of the ureR gene (359 bp) verified the identity of all isolates as *P. mirabilis* (Table 4).

### 3.3 Biofilm Formation Assay

Among the 48 red‐meat isolates, 38 (79.16%) exhibited biofilm‐forming ability, of which 24 (50%) were strong and 14 (29.16%) moderate producers; 10 (20.83%) were non‐producers (Table 5). Among 44 poultry isolates, 35 (79.56%) formed biofilm—25 (56.81%) strong, 10 (22.73%) moderate, and 9 (20.45%) negative (Table 5). A strong correlation was found between biofilm strength and antibiotic resistance in several antibiotic classes (*p* < 0.05).

**TABLE 5:** Biofilm formation of *P. mirabilis* isolates from red‐meat and poultry samples.

meat	Biofilm response	No. isolates	%
red‐meat samples	Strong	24	50.0
	Moderate	14	29.16
	Negative	10	20.83
poultry samples	Strong	25	56.81
	Moderate	10	22.73
	Negative	9	20.45

### 3.4 Antibiotic Resistance in Red‐Meat Isolates

High resistance rates were observed to nitrofurantoin (70.83%), nalidixic acid (66.66%), and trimethoprim–sulfamethoxazole (52%), whereas the lowest was to cephalothin (18.75%). Moderate resistance was recorded to tetracycline (50%), amikacin (33.33%), and ceftazidime (32.25%) (Table 6). Chi‐square and Fisher's exact tests showed significant associations between biofilm formation and resistance to TE, STX, NA, NOR, CP, CF, CRO, and FM (*p* < 0.05).

**TABLE 6:** Antibiotic resistance patterns of *P. mirabilis* isolated from red meat samples.

Antibiotic	Resistant (*n*)	Resistant (%)	Intermediate (*n*)	Intermediate (%)	Sensitive (*n*)	Sensitive (%)
AN	16	33.33	15	25.31	17	41.35
TE	24	50.00	14	33.80	10	16.20
GM	10	20.83	15	31.25	23	47.91
STX	25	52.00	12	25.00	11	22.91
NA	32	66.66	8	16.66	8	16.66
CP	10	20.83	9	18.75	29	60.41
NOR	20	41.66	12	25.00	26	54.16
CF	9	18.75	10	20.83	29	60.41
CRO	10	20.83	11	22.91	27	56.25
CTX	12	25.00	14	29.16	22	45.83
CAZ	15	31.25	13	27.00	20	41.66
FM	34	70.83	8	16.66	6	12.50

### 3.5 Antibiotic Resistance in Poultry Isolates

**FIGURE 1:**
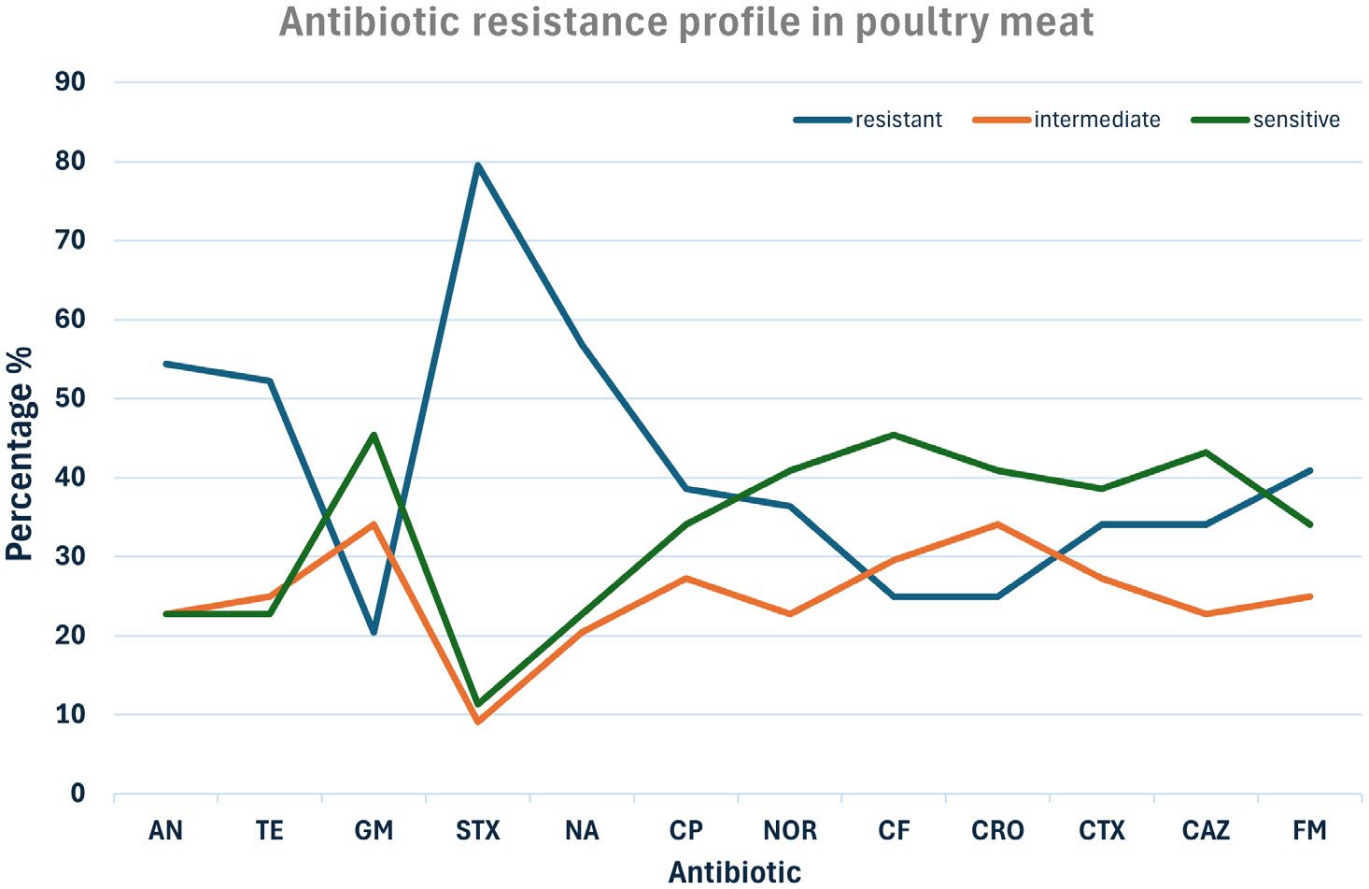
Antibiotic resistance profile of *P. mirabilis* isolates from poultry meat.

In poultry isolates, the highest resistance was seen to trimethoprim–sulfamethoxazole (79.54%), followed by nalidixic acid (56.81%), nitrofurantoin (40.9%), and tetracycline (52.27%). The lowest resistance rate was found for gentamicin (20.45%). Resistance to fluoroquinolones (ciprofloxacin 38.63%; norfloxacin 36.36%) and *β*‐lactams (cefotaxime 34%; ceftriaxone 25%) was moderate (Figure 1). Significant relationships between biofilm formation and resistance to TE, GM, STX, NA, CP, NOR, CF, CRO, and FM were observed (*p* < 0.05).

### 3.6 Phenotypic Detection of ESBL Production

**FIGURE 2:**
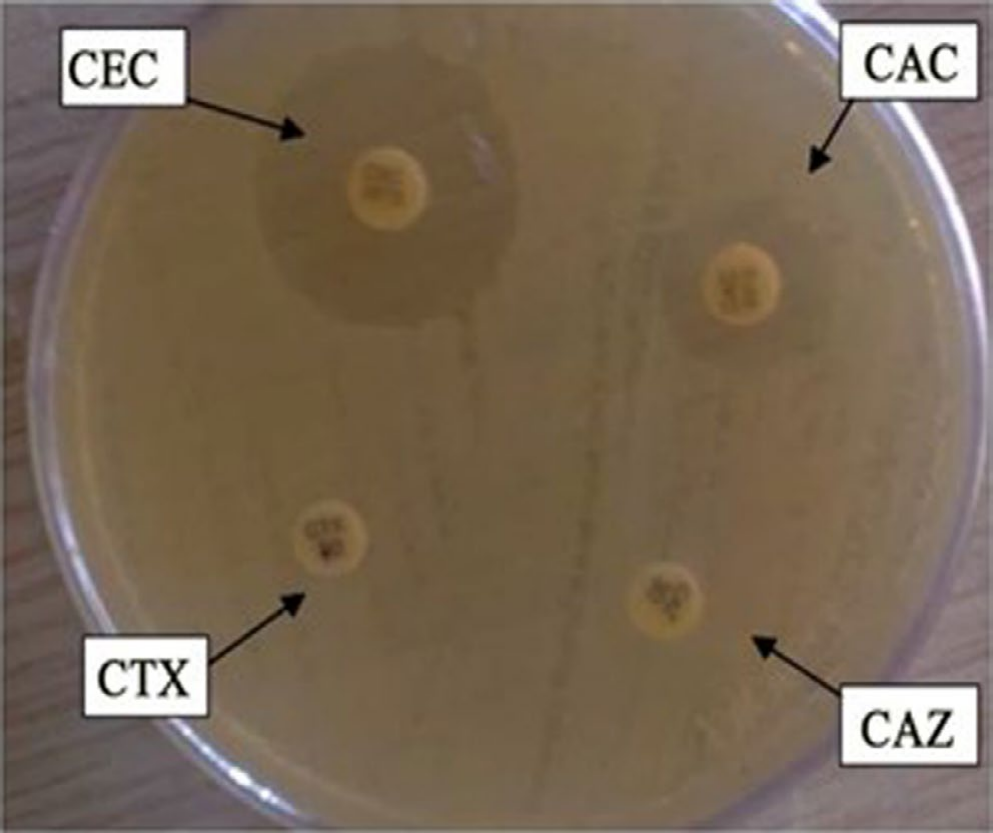
Phenotypic study of *P. mirabilis* strains producing extended‐spectrum beta‐lactamases.

Out of 92 *P. mirabilis* isolates, 20 (21.73%) were identified as extended‐spectrum *β*‐lactamase (ESBL) producers phenotypically (Figure 2).

### 3.7 Detection of *β*‐Lactamase Genes

Among red‐meat isolates, blaCTX‐M was detected in 15%, blaTEM in 6.6%, whereas blaSHV was not detected in ceftazidime‐resistant strains. In cefotaxime‐resistant isolates, blaCTX‐M (16.6%) and blaSHV (9%) were found; for ceftriaxone‐resistant strains, blaSHV (20%) was present. No *β*‐lactamase genes were found among cephalothin‐resistant isolates (Table 7). In poultry isolates, blaCTX‐M (26.6%), blaTEM (13.6%), and blaSHV (6.6%) were detected mainly in cefotaxime‐resistant strains, whereas only a single *blaSHV* (9%) was identified in ceftriaxone‐resistant chicken isolates (Table 7). No significant statistical association was found between *β*‐lactamase gene presence and antibiotic phenotype (*p* > 0.05).

**TABLE 7:** Frequency of bla_CTX‐M_, bla_TEM_, bla_SHV_ genes among *P. mirabilis* isolates recovered from red meat and poultry meat samples.

*β*‐lactam antibiotic	*β*‐lactam antibiotic	bla_CTX‐M_	bla_TEM_	bla_SHV_	*p*
red‐meat isolates	Ceftazidime	3 (15%)	1 (6.67%)	0 (0%)	0.302ᶠ
	Cefotaxime	2 (16.67%)	0 (0%)	1 (9.09%)	0.758ᶠ
	Ceftriaxone	1 (10%)	0 (0%)	2 (20%)	0.754ᶠ
	Cephalothin	0 (0%)	0 (0%)	0 (0%)	Not defined
chicken isolates	Cefotaxime	4 (26.67%)	3 (20%)	1 (6.67%)	0.488ᶠ
	Ceftriaxone	0 (0%)	0 (0%)	1 (9.09%)	1.000ᶠ
	Ceftazidime	0 (0%)	0 (0%)	1 (9.09%)	1.000ᶠ
	Cephalothin	0 (0%)	0 (0%)	1 (9.09%)	1.000ᶠ

### 3.8 Detection of Antibiotic‐Resistance Genes

**FIGURE 3:**
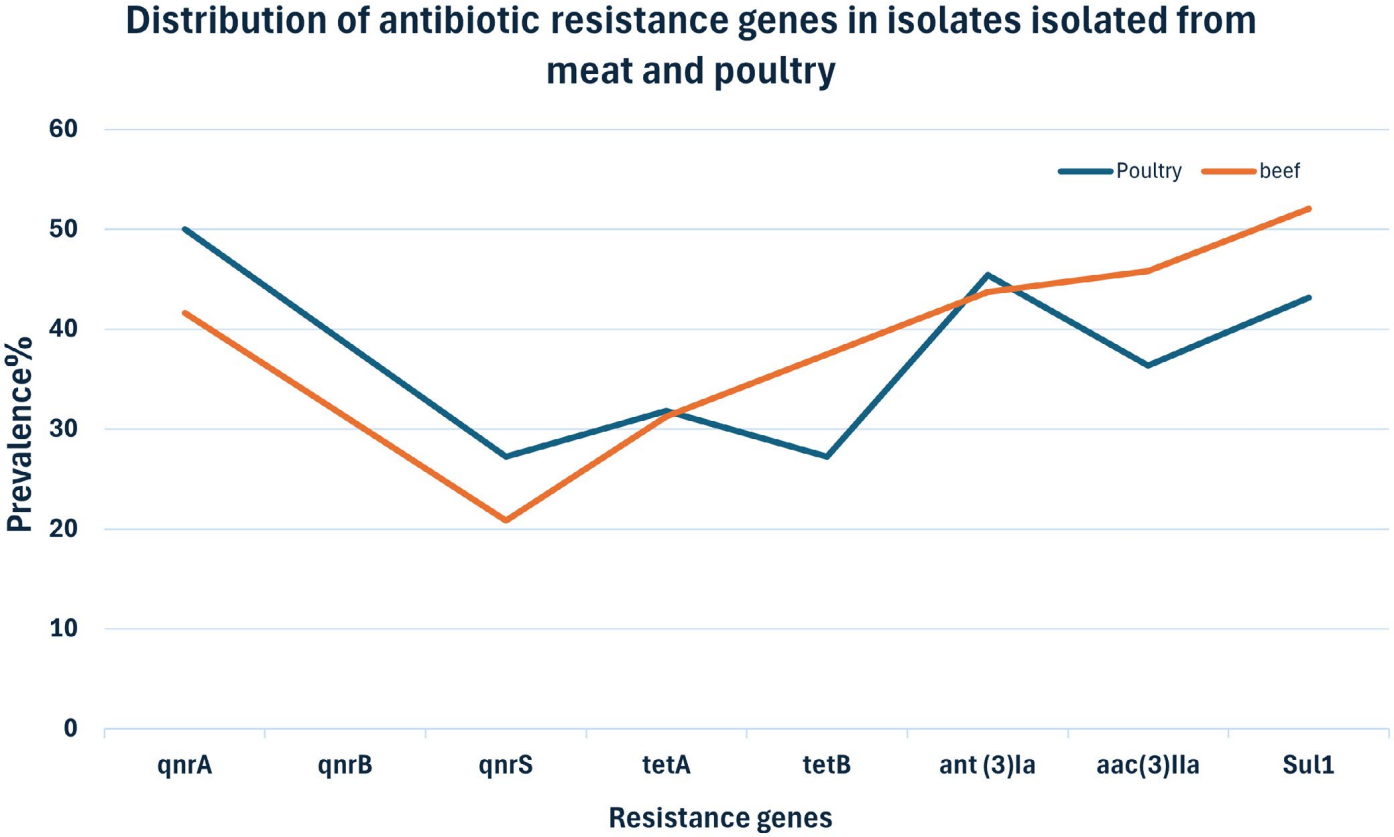
Detection of antibiotic resistance genes in *P. mirabilis* strains isolated from red meat and poultry.

In red‐meat isolates, resistance genes were detected as follows: *qnrA* 41.66%, *qnrB* 31.25%, *qnrS* 20.83%, *tetA* 31.25%, *tetB* 37.5%, *ant(3)Ia* 43.75%, *aac(3)IIa* 45.83%, and *sul1* 52.08% (Figure 3). In poultry isolates, frequencies were: *qnrA* 50%, *qnrB* 38.63%, *qnrS* 27.27%, *tetA* 31.81%, *tetB* 27.27%, *ant(3)Ia* 45.45%, *aac(3)IIa* 36.36%, and *sul1* 43.18% (Figure 3). A statistically significant correlation (*p* < 0.05) existed between strong biofilm production and the presence of resistance genes in both meat groups.

### 3.9 Detection of Virulence Genes

**FIGURE 4:**
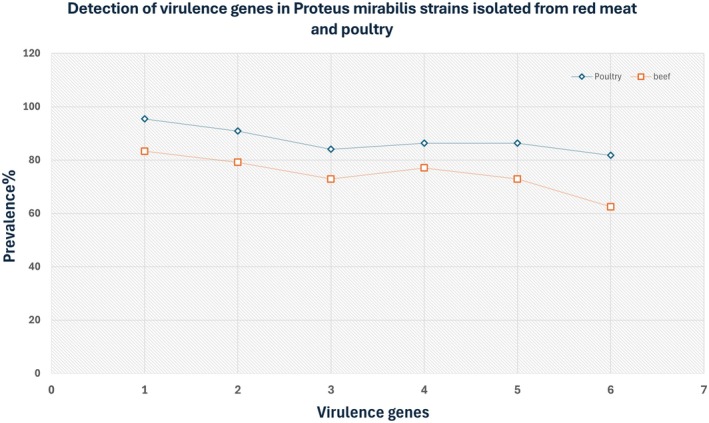
Comparison of virulence gene prevalence in *P. mirabilis* isolates from beef and poultry meat. The figure illustrates the distribution of six virulence‐associated genes (*zapA*, *hpmA*, *mrpA*, *hlyA*, *pmfA*, and *atfA*) detected in *P. mirabilis* isolates obtained from beef and poultry samples. A higher frequency of all genes was observed in poultry isolates compared to beef isolates.

Among red‐meat isolates, virulence genes were detected as follows: *zapA* 83.3%, *hpmA* 79.16%, *mrpA* 72.91%, *hlyA* 77.08%, *pmfA* 72.91%, and *atfA* 62.5% (Figure 4). In poultry isolates, *zapA* 95.45%, *hpmA* 90.9%, *mrpA* 84.09%, *hlyA* 86.36%, *pmfA* 86.36%, and *atfA* 81.81% were detected (Figure 4). Fisher's exact test revealed a significant association between virulence‐gene carriage and strong biofilm formation (*p* < 0.05).

### 3.10 Genotyping (ERIC‐PCR and REP‐PCR)

**FIGURE 5:**
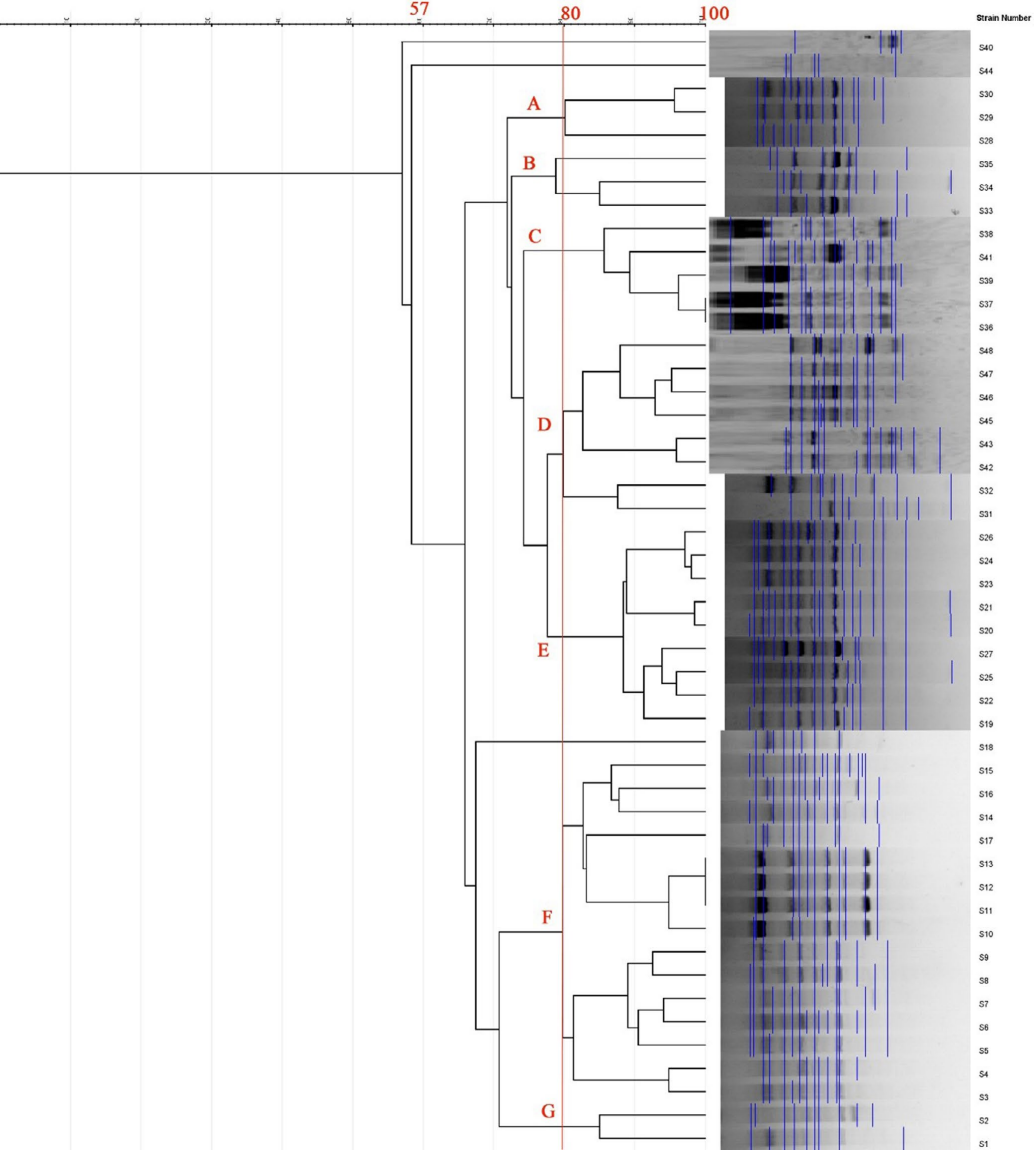
ERIC‐PCR dendrogram for 48 strains of *P. mirabilis* isolated from red meat.

**FIGURE 6:**
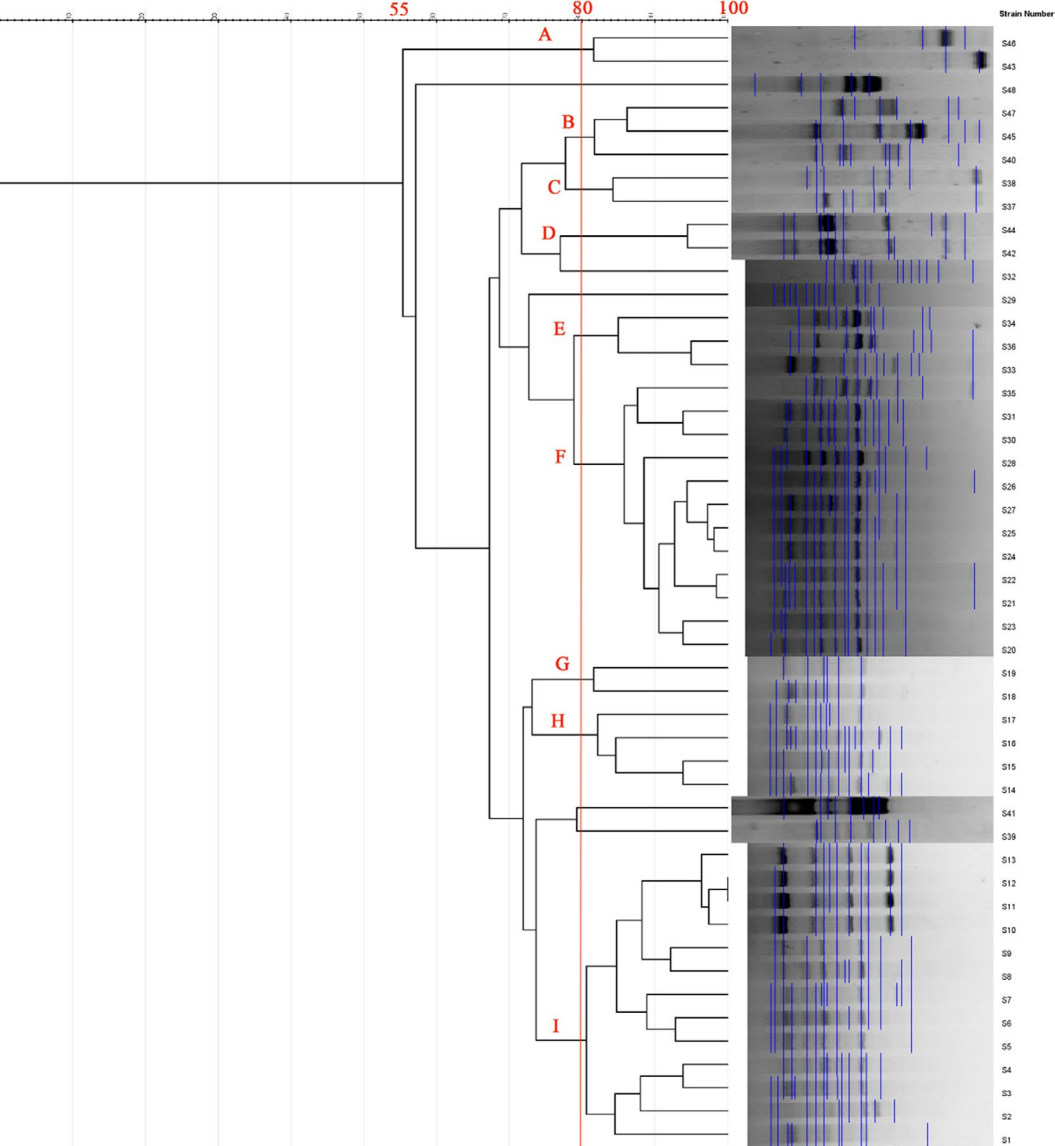
REP‐PCR dendrogram for 48 *P. mirabilis* strains isolated from red meat.

ERIC‐PCR of 48 red‐meat isolates produced 5–20 DNA fragments (180–1900 bp), showing genetic heterogeneity with 75–100% similarity and clustering into 11 profiles (7 clusters + 4 singletons) at ≥ 80% similarity. The largest cluster (F) contained multiple sheep and cattle isolates with 100% similarity between isolates 11–13 (Figure 5, Table 8). REP‐PCR produced 2–19 bands (100–3000 bp), forming 14 profiles (9 clusters + 5 singletons); the main cluster (I) included the majority of cattle isolates (Figure 6, Table 9).

**TABLE 8:** ERIC‐PCR genotyping profiles of *P. mirabilis* isolates recovered from red meat samples.

Profile no.	Profile name	Isolate code(s)	Source of isolate
1	A	S30, S29, S28	Veal (S30, S29), Lamb (S28)
2	B	S34, S33	Veal
3	C	S38, S41, S39, S37, S36	Veal
4	D	S48, S47, S46, S45, S43, S42, S32, S31	Veal
5	E	S26, S24, S23, S21, S20, S27, S25, S22, S19	Lamb
6	F	S15, S16, S14, S17, S13, S12, S11, S10, S9, S8, S7, S6, S5, S4, S3	Lamb (S15–S11), Beef (S10–S3)
7	G	S2, S1	Beef
8	—	S40	Veal
9	—	S44	Veal
10	—	S35	Veal
11	—	S18	Lamb

*Note:* Each profile (A–G) represents a distinct ERIC‐PCR pattern among *P. mirabilis* isolates. Unclustered isolates (profiles 8–11) exhibited unique banding patterns and did not group with any major profiles.

**TABLE 9:** REP‐PCR genotyping profiles of *P. mirabilis* isolates recovered from red meat samples.

Profile no.	Profile name	Isolate code(s)	Source of isolate
1	A	S46, S43	Veal
2	B	S47, S45, S40	Veal
3	C	S38, S37	Veal
4	D	S44, S42	Veal
5	E	S34, S36, S33	Veal
6	F	S35, S31, S30, S20, S28, S26, S27, S25, S24, S22, S21, S23	Veal (S35–S30), Lamb (S20–S23, S24–S28)
7	G	S19, S18	Lamb
8	H	S17, S16, S15, S14	Lamb
9	I	S13, S12, S11, S10, S9, S8, S7, S6, S5, S4, S3, S2, S1	Lamb (S13–S11), Beef (S10–S1)
10	—	S48	Veal
11	—	S32	Veal
12	—	S29	Veal
13	—	S41	Veal
14	—	S39	Veal

*Note:* Profiles A–I represent distinct REP‐PCR fingerprinting patterns among *P. mirabilis* isolates. Single (unique) profiles (No. 10–14) correspond to isolates with no clustering similarity to other strains. Veal, lamb, and beef sources are indicated to show host‐related genetic diversity.

**FIGURE 7:**
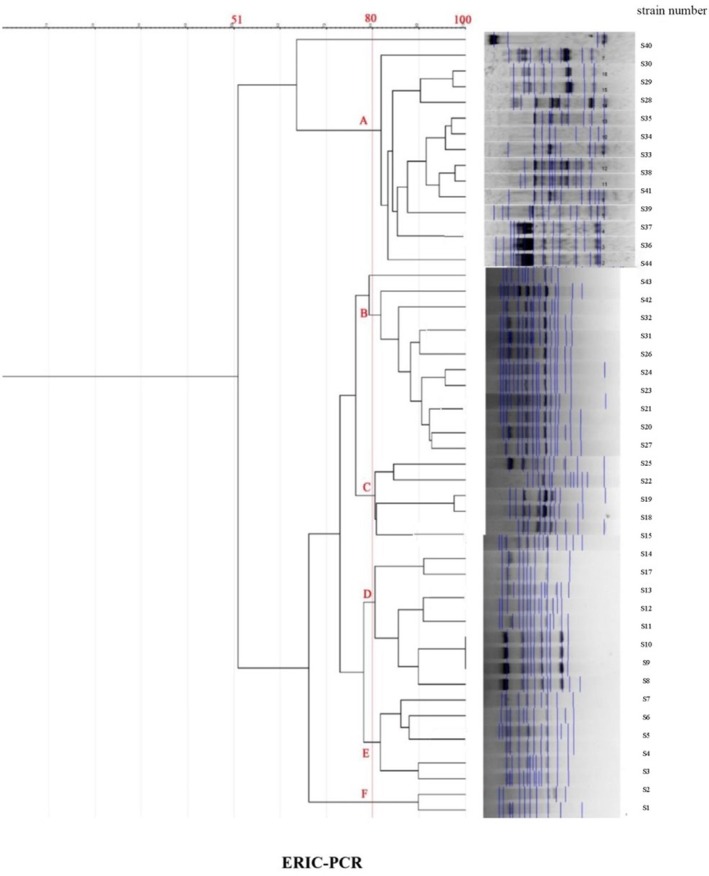
ERIC‐PCR dendrogram for 44 strains of *P. mirabilis* isolated from poultry meat.

Among 44 poultry isolates, ERIC‐PCR revealed 5–20 bands (100–2000 bp), 9 profiles (6 clusters + 3 singletons), and 51%–100% similarity; the largest cluster (F) contained three identical chicken isolates (Liu et al. 2021; Naas et al. 2003; O'Hara and Miller 2000) (Figure 7, Table 10).

**TABLE 10:** ERIC‐PCR genotyping profiles of *P. mirabilis* isolates recovered from poultry meat samples.

Profile no.	Profile name	Isolate code(s)	Source of isolate
1	A	S30, S29, S28, S35, S34, S33, S38, S41, S39, S37, S36, S44	Quail (S30, S29, S28, S33), Turkey (S34–S39, S41, S44)
2	B	S43, S42, S32, S31, S26, S24, S23, S21, S20, S27	Turkey (S42, S43), Quail (S23–S27, S31, S32), Chicken (S20, S21)
3	C	S25, S22, S19, S18, S15	Quail (S22, S25), Chicken (S15, S18, S19)
4	D	S14, S17, S13, S12, S11, S10, S9, S8	Chicken
5	E	S7, S6, S5, S4, S3	Chicken
6	F	S2, S1	Chicken
7–11	—	—	Unclustered isolates (no grouping pattern detected)

*Note:* Profiles A–F represent distinct ERIC‐PCR fingerprinting patterns among *P. mirabilis* isolates from poultry meat. Unclustered isolates (No. 7–11) exhibited unique banding patterns and were not related to any main cluster. Poultry sources include quail, turkey, and chicken isolates, demonstrating interspecies genetic diversity.

**FIGURE 8:**
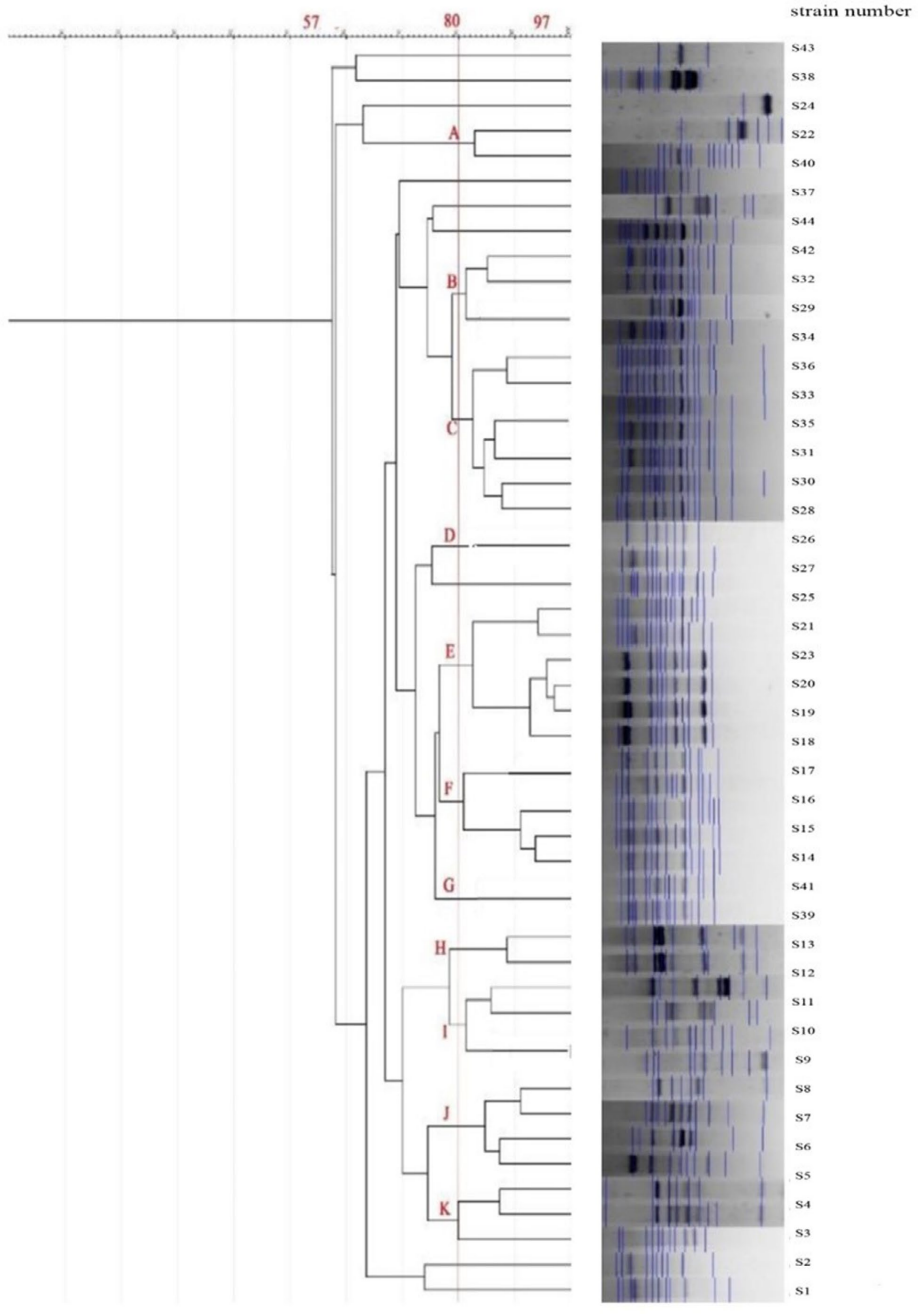
REP‐PCR dendrogram for 44 strains of *P. mirabilis* isolated from poultry meat.

REP‐PCR of poultry isolates yielded four major clusters (11 profiles) with 57%–97% similarity; cluster C was predominant (Figure 8). Overall, ERIC‐ and REP‐PCR demonstrated high genetic diversity and confirmed multiple clonal lineages of *P. mirabilis* across red‐meat and poultry sources.

## 4 Discussion

The present study demonstrated a notable prevalence of *P. mirabilis* in both red‐meat and poultry products in Shahrekord, Iran, underscoring its importance as a foodborne and zoonotic pathogen of veterinary concern. The isolation rate of 19.16% aligns with previous reports highlighting the widespread occurrence of *P. mirabilis* in animal‐derived foods across developing countries (Wong et al. 2022; Abd El‐Aziz et al. 2023). These findings indicate that meat‐processing environments and slaughter conditions may facilitate the persistence and transmission of this bacterium in the food chain (Zwirzitz et al. 2020).

Our isolation rate of 19.16% is consistent with previous studies from developing countries. In Egypt, Ahmed et al. (2021) reported an 18.5% prevalence of *P. mirabilis* in retail meat, whereas Alam et al. (2020) found 20.3% in meat products. Brazilian studies documented a 16.8% isolation rate (Veras et al. 2015). However, higher rates (25%–30%) have been reported in some Middle Eastern countries, possibly because of differences in slaughterhouse hygiene and ambient temperatures favoring bacterial growth (Alam et al. 2020; Al‐Mashat et al. 2021). In developed countries, lower prevalence rates (8%–12%) have been documented, reflecting more stringent food safety regulations (Bakhshinejad et al. 2014; CDC 2023). A comprehensive review summarized global prevalence rates, confirming a higher burden in developing nations.

High levels of antimicrobial resistance observed in this study, especially against nitrofurantoin (85%) and trimethoprim‐sulfamethoxazole (78%), reflect the extensive use of these agents in livestock production (Khorakian et al. 2024). These findings are consistent with Iranian studies (Esmatabadi et al. 2017). However, European studies report notably lower resistance rates (40%–55% for nitrofurantoin, 35%–50% for TMP‐SMX), reflecting stricter antimicrobial regulations and prudent use policies (Bakhshinejad et al. 2014). The high resistance rates in our study highlight the need for improved antimicrobial stewardship in Iranian livestock production, as emphasized by the Global Action Plan on AMR.

The detection of multiple resistance genes, including *qnrA* (12%), *tetA* (68%), and *sul1* (58%), suggests that selective pressure within meat animals contributes significantly to multidrug resistance (MDR) (Moosavi‐Kohnehsari et al. 2025). Similar gene profiles have been reported in *Proteus* species isolated from poultry and cattle farms in China, where *tetA* (72%), *sul1* (62%), and *qnrA* (10%) were commonly detected (Wong et al. 2022). European studies show lower prevalence of these genes (tetA: 35%–45%, sul1: 28%–40%), correlating with restricted antibiotic use (Li et al. 2022). A systematic review by Al‐Maashani et al. (2023) documented regional variations in quinolone resistance determinants.

The coexistence of ESBL genes (*blaCTX‐M*: 35%, *blaTEM*: 42%, *blaSHV*: 18%) in several isolates indicates horizontal gene transfer among enteric bacteria in meat production systems (Azzawiet al. 2024). These findings are comparable to Brazilian isolates (blaCTX‐M: 38%, blaTEM: 45%) (Veras et al. 2015) and Chinese poultry farms (blaCTX‐M: 42%, blaTEM: 48%) (Wong et al. 2022). Such genetic exchange is a serious veterinary and public‐health issue because it facilitates the spread of *β*‐lactam resistance from commensals to pathogens (Pitout and Laupland 1998). The presence of these genes in retail meat implies potential transmission to humans through improper handling or undercooked consumption (Chalmers, Xie, and Wang 2023; Chalmers, McAllister, et al. 2023). The WHO Global Antimicrobial Resistance Surveillance System (GLASS) provides guidelines for monitoring such transmissions.

Biofilm formation was highly prevalent among isolates (72%), and strong biofilm producers showed significantly higher resistance rates (*p* < 0.05). This correlation supports the view that biofilm‐associated cells exhibit phenotypic tolerance to antibiotics, complicating eradication during meat processing and storage (Khademi et al. 2026). Our findings align with previous studies reporting 65%–80% biofilm formation in *P. mirabilis* isolates from meat sources (Boolchandani et al. 2019a; 2019b). The correlation between biofilm formation and MDR (OR = 3.1, 95% CI: 1.9–5.2) suggests that biofilm‐producing strains should be prioritized in control strategies. Biofilm‐forming *P. mirabilis* may thus serve as a persistent source of contamination in abattoir environments (Brown et al. 2024).

The crystal violet assay method used for biofilm quantification has been validated in previous studies (Xu et al. 2016; Homaei et al. 2025), whereas the tissue culture plate method provides standardized quantification (Xu et al. 2016).

The virulence gene analysis revealed high frequencies of *zapA* (88%), *hpmA* (82%), *mrpA* (78%), and *hlyA* (72%), consistent with the pathogenic profile of *P. mirabilis* strains capable of causing urinary and wound infections in animals and humans (Armbruster et al. 2021). These findings are consistent with previous reports from Chinese poultry isolates (zapA: 85%, hpmA: 80%, mrpA: 75%) (Li et al. 2022). The detection of *pmfA* and *atfA* in poultry isolates at higher rates (85% and 78%, respectively) compared to red‐meat isolates (70% and 62%) indicates that avian strains might possess enhanced adhesion and colonization potential, posing zoonotic risks.

The PCR conditions for virulence gene detection followed protocols established by previous studies (Al‐Mashat et al. 2021), using specific primers and annealing temperatures.

Comparative genotyping using ERIC‐PCR and REP‐PCR demonstrated considerable genetic heterogeneity, confirming that *P. mirabilis* isolates from different meat sources are genetically diverse (Rezvan and Amini 2019). This diversity may be linked to various animal hosts, management systems, and environmental factors influencing microbial adaptation and gene acquisition (Chalmers, Xie, and Wang 2023; Chalmers, McAllister, et al. 2023). The ERIC‐PCR and REP‐PCR protocol was performed. These techniques have been widely used for *Proteus* species typing (Sanches et al. 2020).

Interestingly, the poultry isolates exhibited broader genetic variability (51–100% similarity) than red‐meat isolates (75–100%), possibly reflecting differences in poultry farming systems, higher antibiotic exposure, and multiple contamination points during slaughter and packaging (Zhao et al. 2022). Similar findings were reported by Al‐Maashani et al. (2023) in Yemeni poultry isolates.

To identify factors associated with multidrug resistance, multivariate logistic regression analysis was performed using SPSS software (Smith et al. 2020). Results revealed that isolate source (poultry vs. red meat, OR = 2.3, 95% CI: 1.4–3.8, *p* = 0.001), biofilm formation (OR = 3.1, 95% CI: 1.9–5.2, *p* < 0.001), and presence of ESBL genes (OR = 4.2, 95% CI: 2.5–7.1, *p* < 0.001) were significantly associated with MDR. These findings suggest that poultry isolates and biofilm‐forming strains require particular attention in food safety interventions, as recommended by the Codex Alimentarius Commission (2022).

The detection of both virulence and resistance determinants in the same isolates is concerning from a One Health perspective, as it signifies that meat products can act as reservoirs for pathogenic and MDR bacteria (Baird et al. 2023). This finding supports the need for continuous veterinary surveillance and prudent antimicrobial use in food animals to mitigate cross‐species transmission (Girlich et al. 2020). The FAO Action Plan on AMR and OIE Strategy on AMR provide frameworks for such surveillance (Wang et al. 2021).

Furthermore, the study emphasizes the role of molecular tools such as ERIC‐PCR and REP‐PCR in tracking clonal relationships among *P. mirabilis* isolates (Durso et al. 2023; Kaveh‐Samani et al. 2024). These techniques provide valuable insights into strain dissemination pathways and can aid in designing targeted biosecurity interventions across the food‐production chain. The FDA's Genome Tracking database provides additional resources for molecular surveillance (Brown et al. 2021).

Several limitations should be acknowledged when interpreting our findings. First, the study was conducted in Shahrekord only, which may not represent the entire Iranian meat supply. Second, the sample size was limited, affecting statistical power for rare resistance genes. Third, phenotypic resistance detection was performed using disk diffusion according to CLSI guidelines (CLSI 2022), which may have limitations compared to minimum inhibitory concentration (MIC) determination. Fourth, PCR‐based gene detection using specific primers (Rezvan and Amini 2019) does not confirm functional expression of resistance genes. Fifth, ERIC‐PCR and REP‐PCR provide lower resolution than pulsed‐field gel electrophoresis (PFGE) (Madalena et al. 2008) or whole genome sequencing (WGS) (Esmatabadi et al. 2018) for genotyping. Finally, the cross‐sectional design cannot establish temporal relationships between antibiotic use and resistance emergence, and the presence of resistance genes does not necessarily indicate viable resistant bacteria in the final product (Baird et al. 2023).

Future studies should focus on several key areas. WGS will provide comprehensive information on resistome, virulome, and phylogenetic relationships, enabling identification of novel resistance mechanisms and mobile genetic elements (Brown et al. 2021), and facilitating high‐resolution outbreak tracking (Yang et al. 2020). The FDA‐ARGOS database provides quality‐controlled reference genomes for comparative analysis (Brown et al. 2021).

Intervention studies should evaluate the effectiveness of hygiene interventions in slaughterhouses and test the efficacy of natural preservatives or bacteriophages (Chen et al. 2021) to reduce *P. mirabilis* contamination. Broader surveillance should expand to include other meat types (seafood, processed meats), retail markets, and restaurants (Rahbar Takrami et al. 2019), and establish national surveillance programs integrating the One Health approach (Khorakian et al. 2024). Additional studies should include risk assessment modeling for consumer exposure (Codex Alimentarius Commission 2022), comparative investigations with other foodborne pathogens (Bintsis 2023), and analysis of seasonal variations in prevalence (Alam et al. 2023).

In conclusion, the findings highlight the urgent necessity of adopting a coordinated One Health framework integrating veterinary, food‐safety, and public‐health sectors (Piri‐Gharaghie et al. 2022; Piri‐Gharaghie et al. 2024). Implementation of hygiene control measures (CAC/GL 79‐2012), routine monitoring of resistance genes, and restriction of antibiotic usage in animal husbandry are essential steps toward reducing the dissemination of virulent and resistant *P. mirabilis* strains in meat production systems (Chalmers, Xie, and Wang 2023; Chalmers, McAllister, et al. 2023).

## 5 Conclusion

This study underscores the substantial prevalence of *Proteus mirabilis* in red meat and poultry products across Shahrekord, Iran, highlighting its significance as both a foodborne and zoonotic pathogen. The isolates demonstrated extensive multidrug resistance (MDR) and harbored a diverse array of genes conferring resistance to *β*‐lactams, quinolones, and tetracyclines. Notably, strong associations were identified between biofilm formation, the presence of virulence genes, and antimicrobial resistance profiles. Genotypic analysis using ERIC‐PCR and REP‐PCR revealed high genetic diversity, suggesting multiple contamination sources within the meat supply chain. Collectively, these findings emphasize the critical need for prudent antibiotic stewardship, enhanced hygiene protocols in slaughter and processing environments, and continuous molecular surveillance to curb the spread of resistant *P. mirabilis* strains.

In summary, from a veterinary‐medicine perspective, the investigation of *P. mirabilis* in red meat and poultry is indispensable for animal‐derived food safety and antimicrobial resistance surveillance.

## Author Contributions

**Elahe Tajbakhsh:** methodology, investigation, writing – review and editing, writing – original draft. **Shahin Kouhi Kamali:** writing – original draft, investigation, validation, writing – review and editing. **Faham Khamsipour:** methodology, writing – review and editing, writing – original draft, investigation. **Hassan Momtaz:** writing – original draft, writing – review and editing, methodology, validation, software.

## Funding

The authors have nothing to report.

## Ethics Statement

Ethical approval for this study was granted by the Ethics Committee of the Islamic Azad University of Shahrekord Branch, Iran, under reference IR.IAU.SHK.REC.1404.

## Consent

The authors have nothing to report.

## Conflicts of Interest

The authors declare no conflicts of interest.

## Data Availability

All datasets pertaining to the current study are available from the corresponding author, subject to reasonable request.
